# Elevated intracellular cAMP exacerbates vulnerability to oxidative stress in optic nerve head astrocytes

**DOI:** 10.1038/s41419-017-0171-8

**Published:** 2018-02-19

**Authors:** Myoung Sup Shim, Keun-Young Kim, Jung Hyun Bu, Hye Seung Nam, Seung Won Jeong, Tae Lim Park, Mark H. Ellisman, Robert N. Weinreb, Won-Kyu Ju

**Affiliations:** 1 Hamilton Glaucoma Center and Department of Ophthalmology, Shiley Eye Institute, University of California San Diego, La Jolla, CA USA; 2 Center for Research on Biological Systems, National Center for Microscopy and Imaging Research and Department of Neuroscience, University of California San Diego, La Jolla, CA USA

## Abstract

Glaucoma is characterized by a progressive loss of retinal ganglion cells and their axons, but the underlying biological basis for the accompanying neurodegeneration is not known. Accumulating evidence indicates that structural and functional abnormalities of astrocytes within the optic nerve head (ONH) have a role. However, whether the activation of cyclic adenosine 3′,5′-monophosphate (cAMP) signaling pathway is associated with astrocyte dysfunction in the ONH remains unknown. We report here that the cAMP/protein kinase A (PKA) pathway is critical to ONH astrocyte dysfunction, leading to caspase-3 activation and cell death via the AKT/Bim/Bax signaling pathway. Furthermore, elevated intracellular cAMP exacerbates vulnerability to oxidative stress in ONH astrocytes, and this may contribute to axonal damage in glaucomatous neurodegeneration. Inhibition of intracellular cAMP/PKA signaling activation protects ONH astrocytes by increasing AKT phosphorylation against oxidative stress. These results strongly indicate that activation of cAMP/PKA pathway has an important role in astrocyte dysfunction, and suggest that modulating cAMP/PKA pathway has therapeutic potential for glaucomatous ONH degeneration.

## Introduction

Primary open-angle glaucoma (POAG) is characterized by a slow and progressive degeneration of retinal ganglion cells (RGCs) and their axons in the optic nerve (ON), leading to loss of visual function^1^. The factors contributing to axon degeneration in the optic nerve head (ONH) in POAG are not well understood. Accumulating evidence indicates that astrocyte dysfunction accompanied by RGC axon loss is closely associated with the pathogenesis of glaucomatous ONH degeneration^2–4^. Indeed, structural and functional abnormalities of astrocytes have been reported in the ONH of experimental glaucoma models as well as patients with POAG^2,3,5,6^.

The ubiquitous second messenger cyclic adenosine 3′,5′-monophosphate (cAMP) in the central nervous system contributes to numerous biological processes including cell growth and death^7–10^. Upon stimulation, cAMP synthesis and its degradation are tightly regulated by adenyl cyclases (ACs) and cyclic nucleotide phosphodiesterases (PDEs), respectively^8^. Previous studies have demonstrated that the basal level of cAMP was significantly higher in the unstimulated glaucomatous ONH astrocytes from Caucasian American and African American (AA) donors with POAG compared with their unstimulated ONH astrocytes from normal healthy counterparts^11^. Furthermore, elevated hydrostatic pressure, a mimetic of high intraocular pressure (IOP) *in vitro*, upregulated the mRNA expression of two transmembrane ACs (tmACs) genes, *ADCY3* (*AC3*), and *ADCY9* (*AC9*), in the ONH astrocytes from AA donors^12^. This suggests an intriguing possibility that the activation of intracellular cAMP signaling pathway may have a role in the pathogenesis of glaucomatous ONH astrocytes. Nevertheless, the precise cellular and molecular mechanism(s) of the activation of cAMP signaling pathway in astrocyte dysfunction in glaucomatous ONH degeneration remains to be determined. These mechanisms may be related to oxidative stress.

Oxidative stress has long been thought to be an important pathophysiological mechanism in many neurodegenerative diseases including glaucoma^2,4,13–15^. Astrocytes are the responsible cell type for many pathological alterations in the oxidative stress-mediated glaucomatous ONH degeneration^2–4,16^. Interestingly, activation of the tmACs-mediated cAMP/protein kinase A (PKA) signal pathway induced by forskolin is associated with increased vulnerability to hydrogen peroxide (H_2_O_2_)-induced oxidative stress in rat neocortical astrocytes *in vitro*^17^. These findings collectively suggest that the elevated intracellular cAMP-mediated PKA signaling pathway contributed to oxidative stress-induced dysfunction of astrocytes during glaucomatous ONH degeneration.

In the present study, we investigate the impact of activation of cAMP/PKA pathway and its synergistic effect with oxidative stress on ONH astrocyte dysfunction.

## Results

### Elevated cAMP impairs ONH astrocytes, but promotes RGC survival

**Fig. 1:**
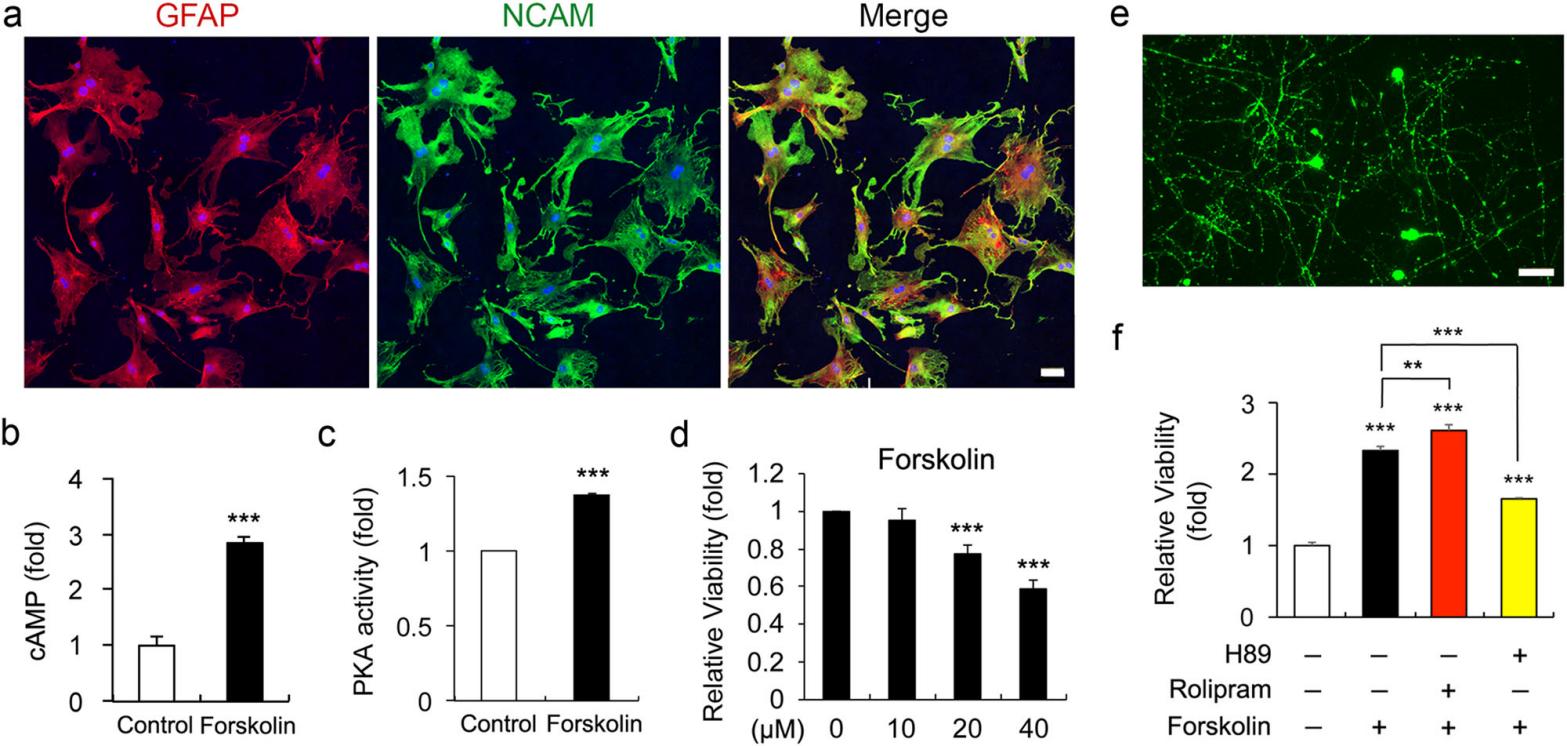
Effects of elevated intracellular cAMP on ONH astrocytes and RGCs **a** Immunocytochemial analysis of GFAP and NCAM immunoreactivity in purified ONH astrocytes. Scale bar, 20 μm. **b** Intracellular cAMP level ONH astrocytes treated with forskolin (10 μM) for 1 h. **c** PKA activity in ONH astrocytes treated with forskolin (10 μM) for 1 h. **d** Cell viability analysis using MTT assay in ONH astrocytes treated with forskolin (0–40 μM) for 1 h. **e** Thy-1 (green)-positive primary RGCs cultures. Scale bar, 20 μm. **f** Cell viability assay using MTT in cultured RGCs treated with forskolin (10 μM), rolipram (2 μM), or H89 (10 μM) for 48 h. For each determination, the cAMP level was normalized to the protein contents, and the PKA activity and cell viability in controls was normalized to a value of 1.0. Data are shown as the mean ± S.D. (*n* = 3). **P < 0.01; ***P < 0.001 (two-tailed unpaired Student’s *t*-test)

Intriguingly, our results demonstrated that forskolin treatment significantly increased intracellular cAMP level and PKA activity, accompanying by dose-dependent reduction of cell viability measured by 3-[4, 5-dimethylthiazol-2yl]-2, 5-diphenyl tetrazolium bromide (MTT) assay (Figs. 1a-d). As it has been reported that AC and PDE4 (a cAMP-specific PDE) are two key enzymes that control intracellular cAMP level in RGCs^18^, we co-treated RGCs with forskolin and rolipram, a specific inhibitor of PDE4, to enhance cAMP-mediated PKA activity and with forskolin and H89 to inhibit cAMP-mediated PKA activity. The elevated cAMP level was positively correlated with viability of RGCs (Fig. 1e), whereas PKA inhibition significantly suppressed cAMP-mediated promotion of cell viability in cultured RGCs (Fig. 1f).

### Elevated cAMP inhibits AKT phosphorylation and induces caspase-3-mediated cell death in ONH astrocytes

**Fig. 2:**
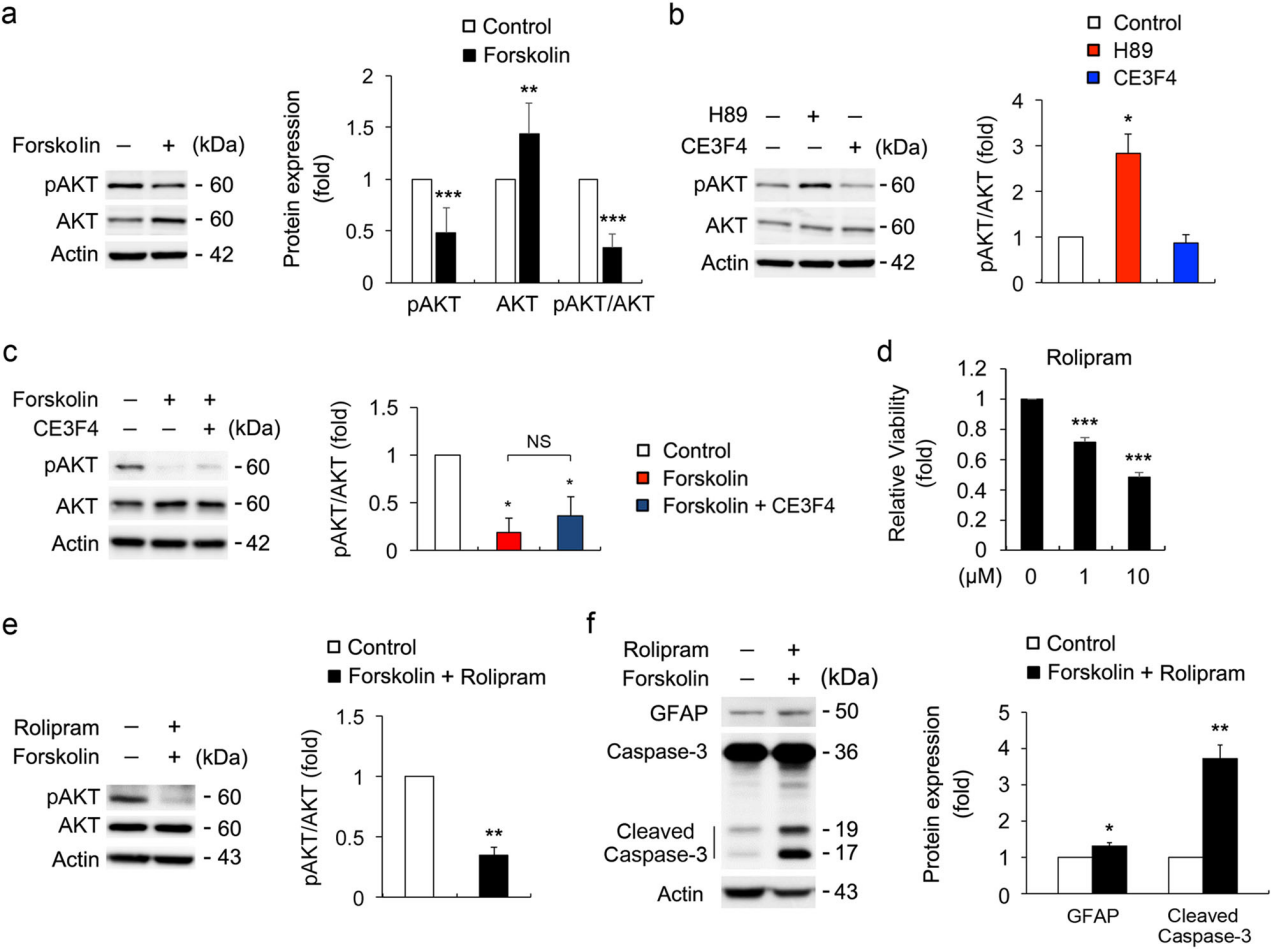
Effects of elevated intracellular cAMP on AKT phosphorylation and caspase-3 protein expression in ONH astrocytes **a** Immunoblot analyses of pAKT S473 and total AKT in ONH astrocytes treated with forskolin (10 μM) for 1 h. **b** Immunoblot analyses of pAKT S473 and total AKT in ONH astrocytes treated with H89 (10 μM) or CE3F4 (10 μM) for 1 h. **c** Immunoblot analyses of pAKT S473 and total AKT in ONH astrocytes treated with CE3F4 (10 μM) and/or forskolin (10 μM) for 1 h. **d** Cell viability assay in rolipram-treated ONH astrocytes for 1 h. **e** Immunoblot analysis of pAKT S473 and total AKT in ONH astrocytes co-treated with forskolin (10 μM) and rolipram (2 μM) for 24 h. **f** Immunoblot analysis of GFAP and caspase-3 in ONH astrocytes treated with forskolin (10 μM) and rolipram (2 μM) for 24 h. Note that enhanced cAMP level induced caspase-3 mediated cell death in ONH astrocytes. For each determination, the cell viability as well as the ratio of pAKT/total AKT protein level and other protein expression in controls was normalized to a value of 1.0. Data are shown as the mean ± S.D. (*n* = 3). *P < 0.05; **P < 0.01; ***P < 0.001 (two-tailed unpaired Student’s *t*-test)

Elevated cAMP significantly decreased the level of AKT phosphorylation (pAKT) at serine 473 (S473) normalized by total AKT in ONH astrocytes (Fig. 2a). Using forskolin together with H89, a PKA inhibitor, or CE3F4, a specific inhibitor of an exchange protein directly activated by cAMP (Epac)^19^, we further addressed that elevated cAMP-mediated inhibition of AKT phosphorylation was dependent on the PKA pathway but not Epac pathway (Figs. 2b and c). Following prolonged elevation of cAMP levels by forskolin and rolipram (24 h), the polygonal, fibroblast-like shape of ONH astrocytes were changed to the extensive stellate-like morphology within a few hours, most likely to be a reactive astrogliosis and this morphological alteration was sustained for 24 h upon increasing cAMP level (Supplementary Fig. S1). Notably, consistent with compromised cell viability in ONH astrocytes by the rolipram treatment (1 h; Fig. 2d), prolonged elevation of cAMP level not only suppressed AKT phosphorylation but also increased the cleaved caspase-3 protein level in ONH astrocytes (Figs. 2e and f).

### Inhibiting cAMP/PKA pathway prevents Bim/Bax pathway and caspase-3 activation in ONH astrocytes

**Fig. 3:**
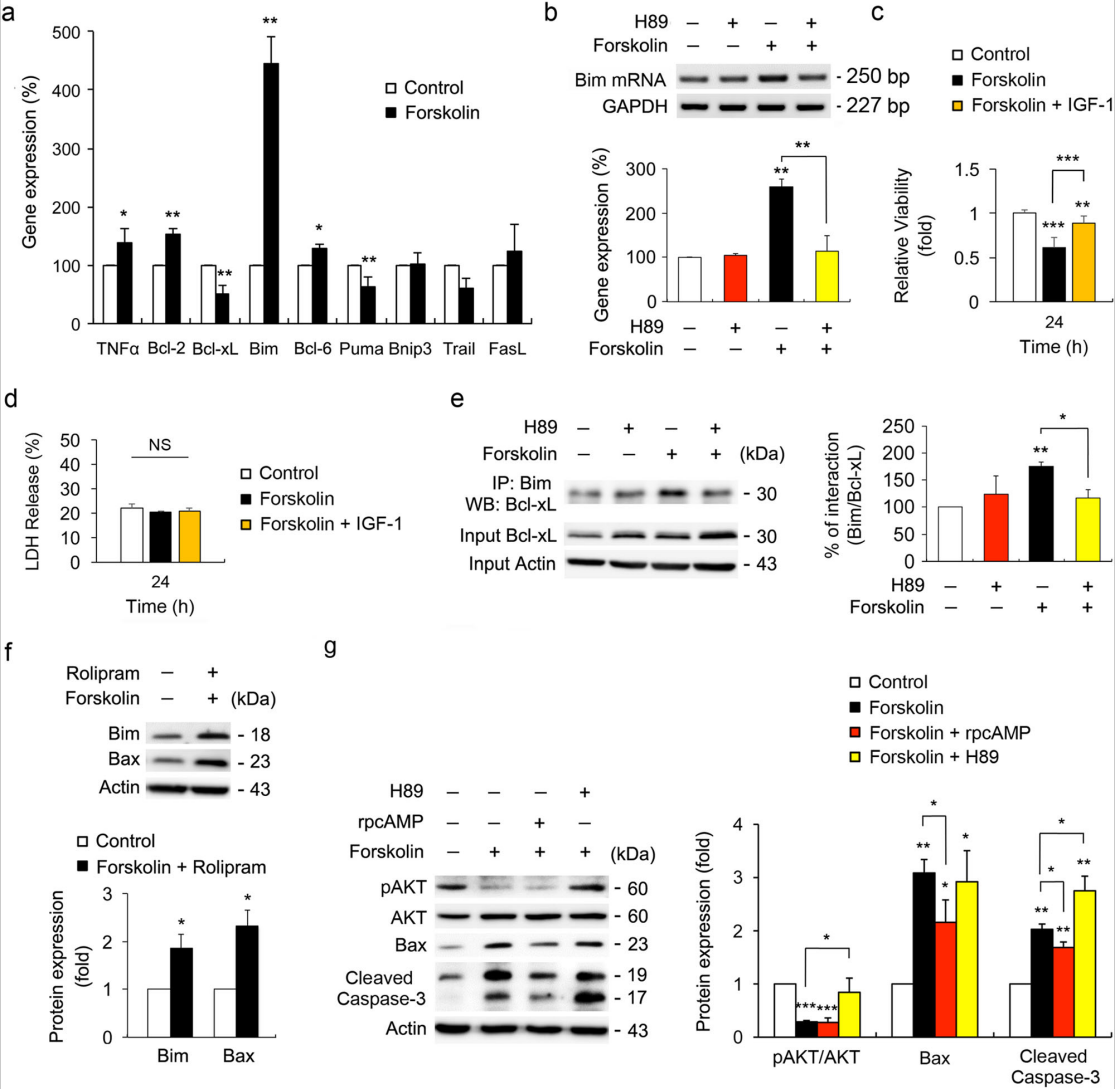
Activation of cAMP/PKA pathway induces ONH astrocytes death via Bax and caspase-3 activation **a** Real-time RT-PCR analysis of NFκB- and FoxO-dependent gene expression in ONH astrocytes treated with forskolin (10 μM) for 1 h. Data were normalized by GAPDH expression. **b** Real-time RT-PCR analysis of Bim mRNA expression in ONH astrocytes treated with forskolin (10 μM) or forskolin (10 μM) plus H89 (10 μM) for 1 h. Note that PKA dependent-regulation of Bim mRNA expression. **c** and **d** Cell viability and cell death analysis using MTT assay (**c**) and LDH assay (d) in ONH astrocytes co-treated with forskolin (10 μM) and IGF-1 (100 nM) for 24 h. **e** Co-IP analysis of Bim and Bcl-xL interaction in ONH astrocytes treated with forskolin (10 μM), H89 (10 μM) or forskolin (10 μM) plus H89 (10 μM) for 1 h. **f** Immunoblot analysis of Bim and activated Bax in ONH astrocytes co-treated with forskolin (10 μM) and rolipram (2 μM) for 24 h. **g** Immunoblot analyses of pAKT, AKT, activated Bax and caspase-3 in ONH astrocyte treated with forskolin (10 μM), Rp-cAMP (50 μM), or H89 (10 μM) for 24 h. For each determination, the mRNA, ratio of pAKT/AKT protein and other protein expression in controls was normalized to a value of 100% or 1.0. Data are shown as the mean ± S.D. (*n* = 3). *P < 0.05; **P < 0.01; ***P < 0.001 (two-tailed unpaired Student’s *t*-test)

Quantitative real-time reverse transcription (RT)-polymerase chain reaction (PCR) analysis demonstrated that forskolin treatment induced a substantial increase of Bim expression in ONH astrocytes among apoptosis-related genes that are regulated by NF-κB (TNFα, Bcl-2, and Bcl-xL) or FoxO (Bim, Bcl-6, Puma, Bnip3, Trail, and FasL) (Fig. 3a). By selecting Bim as an AKT target gene regulated by cAMP, we further observed that PKA inhibition by H89 significantly suppressed Bim mRNA expression in forskolin-treated ONH astrocytes (Fig. 3b). Insulin-like growth factor 1 (IGF-1) regulates apoptosis and oxidative stress response via phosphatidylinositol-5-bisphosphate 3-kinase (PI3K)/AKT signaling pathway in astrocytes^20^. We found that IGF-1 reversed cell viability that was reduced by forskolin treatment alone in an AKT-FoxO-dependent manner in ONH astrocytes (Fig. 3c and Supplementary Fig. S2). There was no significant difference in lactate dehydrogenase (LDH) release between forskolin- and IGF-1-treated ONH astrocytes (Fig. 3d).

Co-immunoprecipitation (Co-IP) revealed that forskolin treatment promoted the interaction between Bim and Bcl-xL in ONH astrocytes (Fig. 3e). In contrast, PKA inhibition blocked this interaction in forskolin-treated ONH astrocytes (Fig. 3e). Furthermore, prolonged elevation of cAMP levels by forskolin and rolipram (24 h) significantly increased both Bim and activated Bax protein expression in ONH astrocytes (Fig. 3f). By co-treatment with forskolin, Rp diastereomers of cAMP (Rp-cAMP), a PKA inhibitor, or H89 in cultured ONH astrocytes for 24 h, inhibition of cAMP/PKA pathway by Rp-cAMP significantly decreased activated Bax and cleaved caspase-3 protein expression in ONH astrocytes, whereas the expression level of pAKT S473 in ONH astrocytes was not changed compared with the control treated with forskolin (Fig. 3g). However, interestingly, inhibition of cAMP/PKA pathway by H89 significantly increased the levels of pAKT S473, whereas cleaved caspase-3 protein expression was not decreased and the expression level of Bax in ONH astrocytes was not changed (Fig. 3g). In addition, consistent with our result of reduction in activated Bax and caspase-3 protein expression, immunocytochemical analysis confirmed that inhibition of cAMP/PKA pathway by Rp-cAMP decreased immunoreactivities for activated Bax and caspase-3 protein expression that were increased in forskolin-treated ONH astrocytes, accompanied by an extensive stellate-like morphology that is likely to be a reactive astrogliosis (Supplementary Fig. S3).

### Enhanced cAMP levels are correlated with an increase in activated Bax and caspase-3 expression in glaucomatous ONH astrocytes

**Fig. 4:**
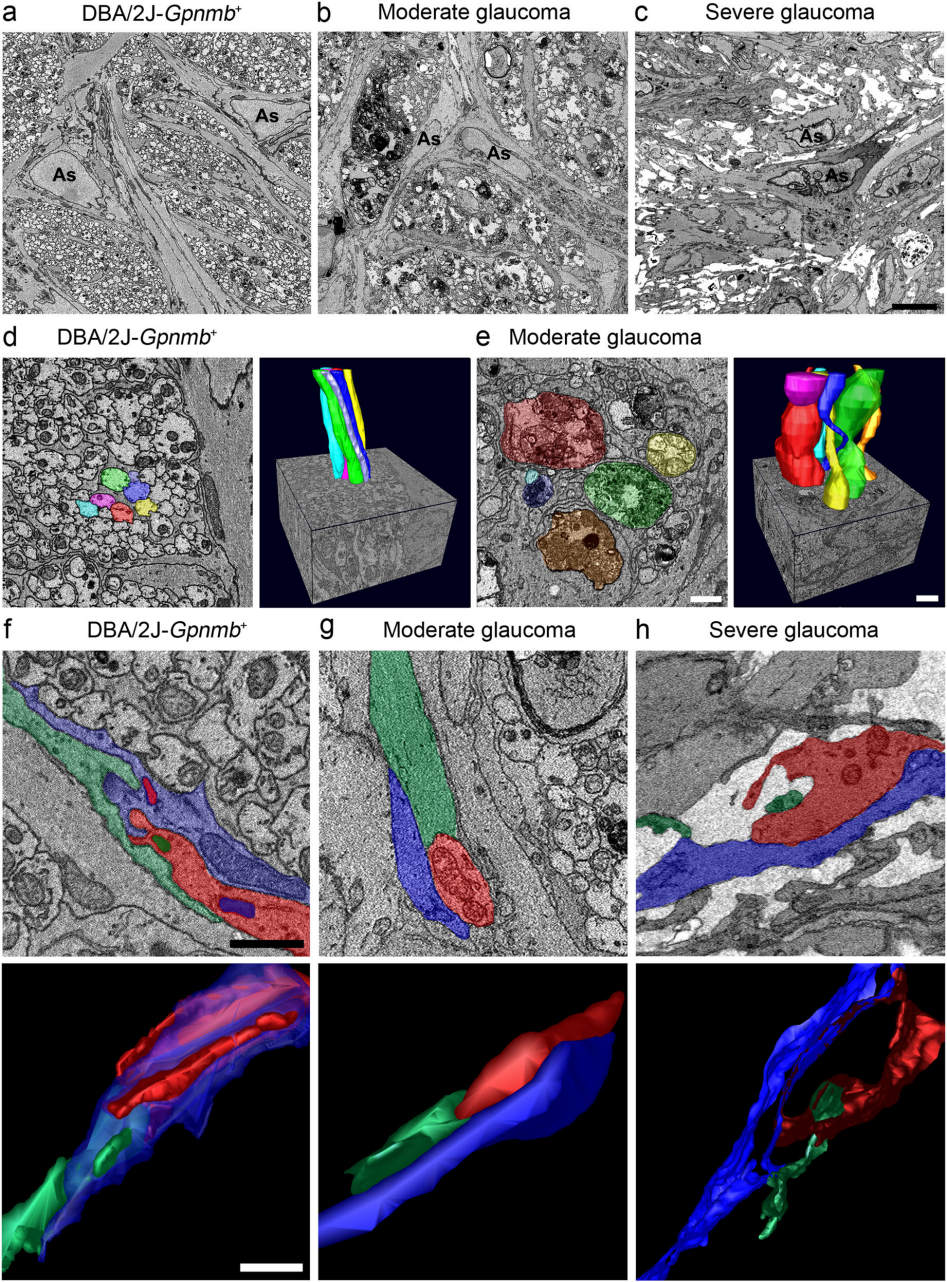
Axon degeneration and structural impairment of astrocyte processes in the glial lamina of glaucomatous DBA/2 J mice **a** Representative SBEM micrographs showed normal healthy morphology of astrocytes and axons in the glial lamina of aged matched non-glaucomatous DBA/2J-*Gpnmb+*mice. **b** Representative SBEM micrographs showed abnormal morphology of astrocytes and degenerative axons in the glial lamina of 10-month-old glaucomatous DBA/2 J mice that have moderate damage. **c** Representative SBEM micrographs showed degenerative astrocytes and severe loss of axons in the glial lamina of glaucomatous DBA/2 J mice that has severe damage. Scale bar, 20 nm. **d** and **e** Axonal segmentation from SBEM volumes. Representative 3D reconstruction micrographs showed normal healthy morphology of axons with various colors in the glial lamina of non-glaucomatous DBA/2J-*Gpnmb+*mice **d**. Representative 3D reconstruction micrographs showed abnormal morphology of axons with various colors in the glial lamina of glaucomatous DBA/2 J mice that have moderate damage **e**. **f**–**h** Astrocyte process segmentation from SBEM volumes. Representative 3D reconstruction micrographs showed normal healthy morphology of astrocyte processes with various colors in the glial lamina of non-glaucomatous DBA/2J-*Gpnmb+*mice **f**. Note the arrangement of astrocyte processes, which is likely to show a shape of hook and loop. Representative 3D reconstruction micrographs showed a loosen arrangement of astrocyte processes with various colors in the glial lamina of glaucomatous DBA/2 J mice that have moderate damage **g**. Representative 3D reconstruction micrographs showed loss of astrocyte processes with various colors in the glial lamina of glaucomatous DBA/2 J mice that have severe damage **h**. Scale bar, 20 μm

**Fig. 5:**
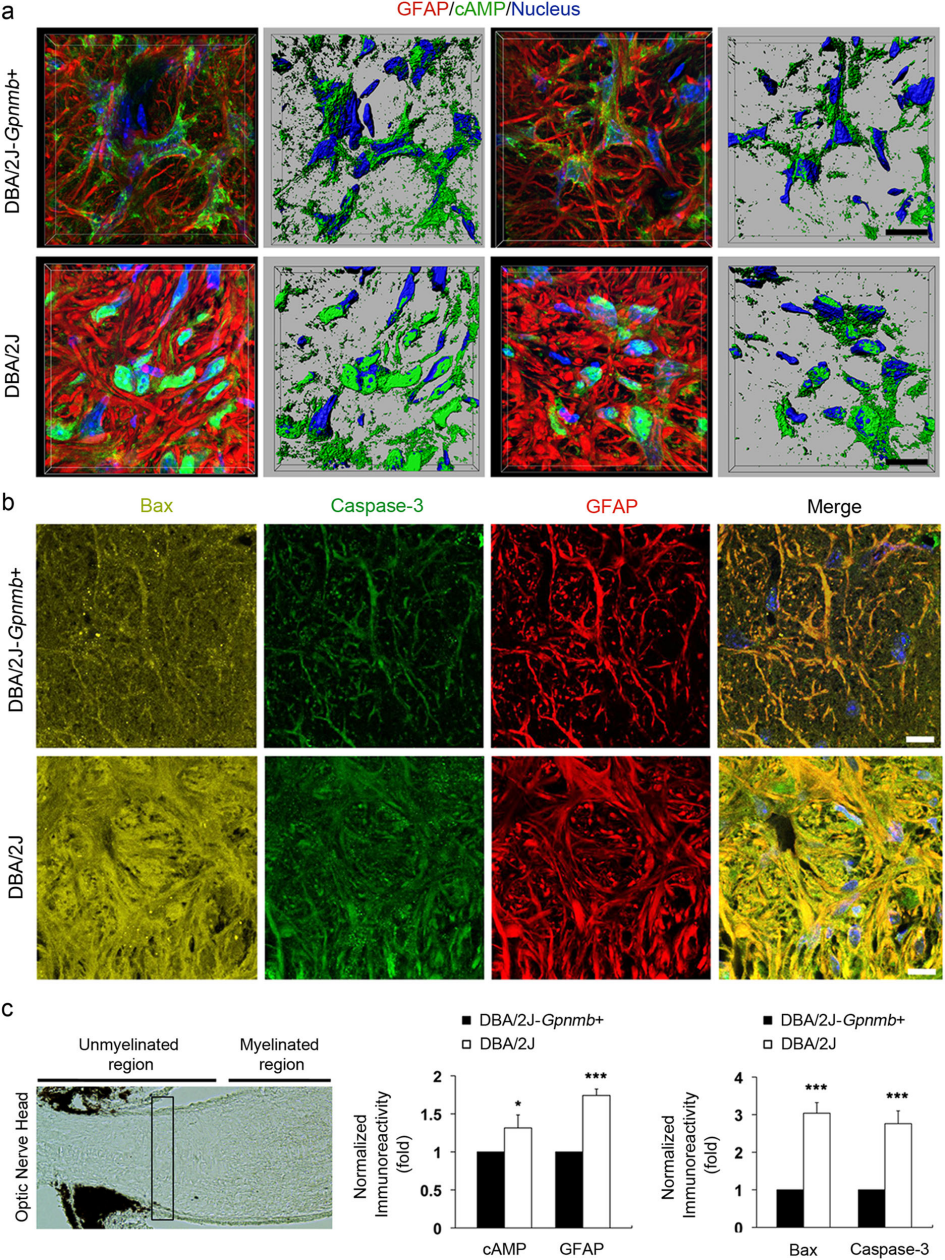
Increased expression of cAMP as well as activated Bax and caspase-3 in astrocytes of the glial lamina in glaucomatous DBA2/J mice **a** and **b** Immunohistochemical analyses of cAMP (green), activated Bax (yellow), and caspase-3 (green) as well as GFAP (red) immunoreactivity in the ONH astrocytes in the glial lamina of age-matched non-glaucomatous DBA/2J-*Gpnmb*^*+*^ and 10-month-old glaucomatous DBA/2 J mice. Note that representative images showed increases of cAMP, activated Bax, and caspase-3 immunoreactivity in astrocytes of the glial lamina in glaucomatous DBA/2 J mice compared with non-glaucomatous DBA/2J-*Gpnmb*^*+*^. Nuclei were stained with Hoechst 33342. Scale bar, 20 μm. **c** Quantitative analysis showed significant increases of cAMP and GFAP, as well as activated Bax and caspase-3 immunoreactivity in glaucomatous DBA/2 J mice. For each determination, the immunoreactivity in controls was normalized to a value of 1.0. Data are shown as the mean ± S.D. (*n* = 5). *P < 0.05; ***P < 0.001 (two-tailed unpaired Student’s *t*-test)

Because glaucomatous DBA/2 J mice, an extensively characterized strain that spontaneously develops elevated IOP with aging^21^, show the structural and functional abnormalities of ONH astrocytes in glaucoma progression^3,5,6^, we used DBA/2 J mice to determine whether glaucomatous ONH astrocytes alters the expression levels of cAMP, Bax and caspase-3 protein. Serial block-face scanning electron microscopy (SBEM) data sets showed degenerative structural changes in astrocyte processes in the glial lamina of 10-month-old glaucomatous DBA2/J mice, accompanied by axon degeneration (Figs. 4a–c). Further analysis of three-dimensional reconstruction of segmentations of axon bundle and astrocyte processes in SBEM stacks revealed a well-organized longitudinal shape of axon bundle in the glial lamina of DBA/2J-*Gpnmb*^*+*^ mice (Fig. 4d), whereas distorted axon bundles were prominent in the glial lamina of glaucomatous DBA/2 J mice (Fig. 4e). On the other hand, astrocytes in the glial lamina of DBA/2J-*Gpnmb*^*+*^ mice presented an irregular arrangement of processes, which is likely a shape of hook and loop (Fig. 4f and Supplementary Movie S1). However, although a moderate glaucoma damage caused a loosen arrangement of astrocytic processes (Fig. 4g and Supplementary Movie S2), severe glaucoma damage induced a significant loss of astrocytic processes in the glial lamina of glaucomatous DBA/2 J mice (Fig. 4h and Supplementary Movie S3). Importantly, glaucomatous astrocytes showed an increase of cAMP, Bax, and caspase-3 immunoreactivities (Figs. 5a–c and Supplementary Fig. S4).

### Elevated cAMP exacerbates vulnerability to oxidative stress in ONH astrocyte dysfunction

**Fig. 6:**
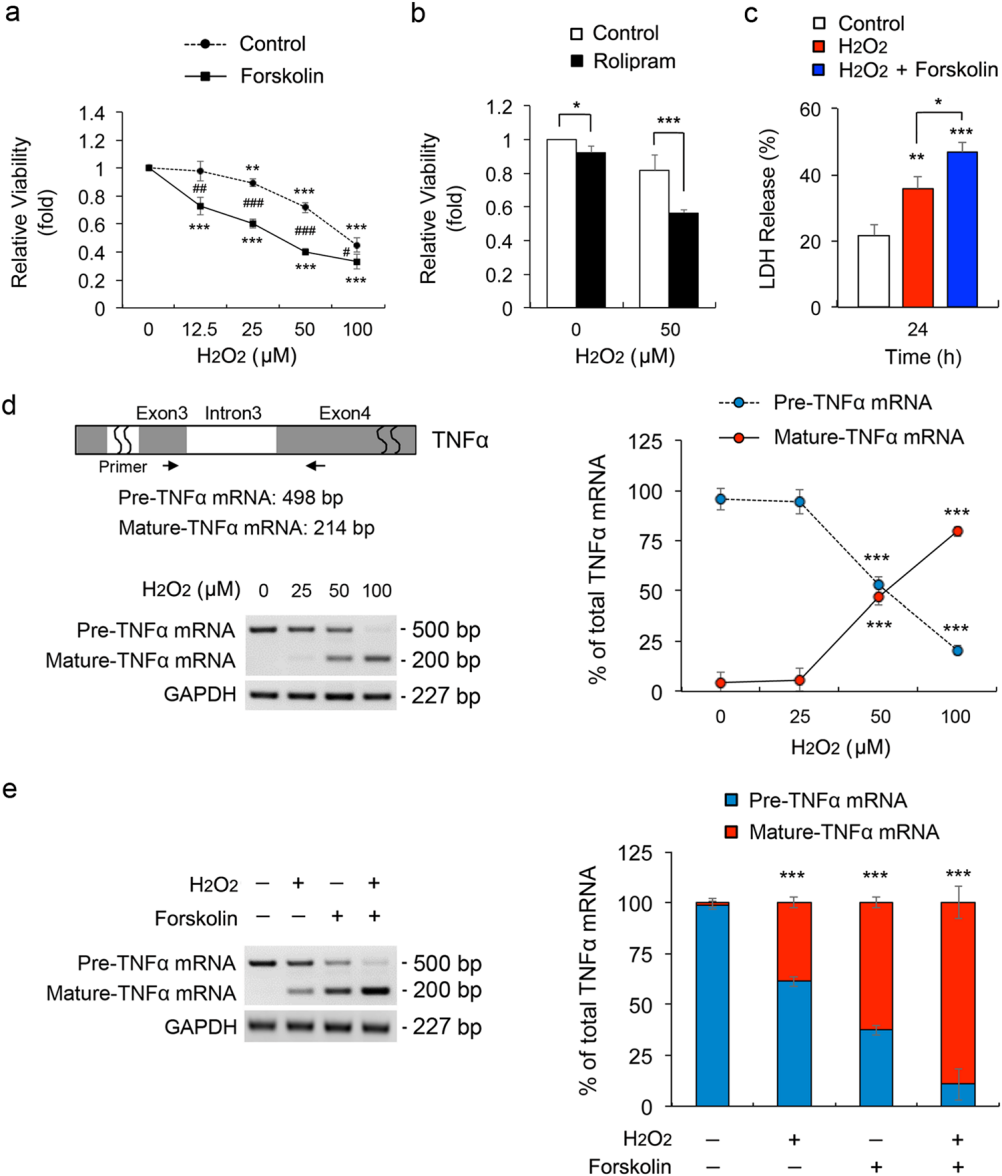
Elevated intracellular cAMP exacerbates vulnerability to oxidative stress in ONH astrocytes **a** Cell viability assay in ONH astrocytes treated with H_2_O_2_ (50 μM) and/or forskolin (10 μM) for 1 h. **b** Cell viability analysis using MTT assay in ONH astrocytes treated with H_2_O_2_ (50 μM) and/or rolipram (2 μM) for 1 h. **c** Cell death analysis using LDH assay in ONH astrocytes treated with H_2_O_2_ (50 μM) and/or forskolin (10 μM) for 24 h. **d** Schematic illustration of rat pre-TNFα mRNA structure for RT-PCR analysis. The primer location was indicated by arrows. RT-PCR analysis of TNF-α splicing in ONH astrocytes treated with H_2_O_2_ (0–100 μM) for 1 h. **e** RT-PCR analysis of TNF-α splicing in ONH astrocytes treated with H_2_O_2_ (50 μM) and/or forskolin (10 μM) for 1 h. Data were normalized by GAPDH expression. For each determination, the cell viability, cell death and mRNA expression in controls was normalized to a value of 1.0 or 100%. Data are shown as the mean±S.D. (*n* = 3). *P < 0.05; **P < 0.01; ***P < 0.001 (two-tailed unpaired Student’s *t*-test)

Of interest, elevated intracellular cAMP by forskolin exacerbated the reduction of cell viability in oxidative stress-induced ONH astrocytes by a dose-dependent manner (Fig. 6a) and enhanced cAMP level by rolipram significantly decreased cell viability in oxidative stress-induced ONH astrocytes compared with controls (Fig. 6b). Furthermore, elevated cAMP significantly increased cell death in oxidative stress-induced ONH astrocytes (Fig. 6c). The splicing of the precursor TNFα mRNA into the mature form is responsive to external stresses^22^. Quantitative RT-PCR analyses demonstrated that H_2_O_2_ treatment alone induced a significant increase of the expression level of mature *TNFα* mRNA in ONH astrocytes in a dose-dependent manner, in contrast to a significant decrease in the expression level of precursor transcripts of TNFα mRNA (Fig. 6d). Interestingly, forskolin treatment alone also induced the reciprocal shifts in the expression levels of precursor and mature *TNFα* mRNA in ONH astrocytes by decreasing precursor *TNFα* mRNA but by increasing mature *TNFα* mRNA (Fig. 6e). More importantly, forskolin treatment in H_2_O_2_-treated ONH astrocytes induced the maximum shift from the precursor to mature *TNFα* mRNA level (Fig. 6e).

**Fig. 7:**
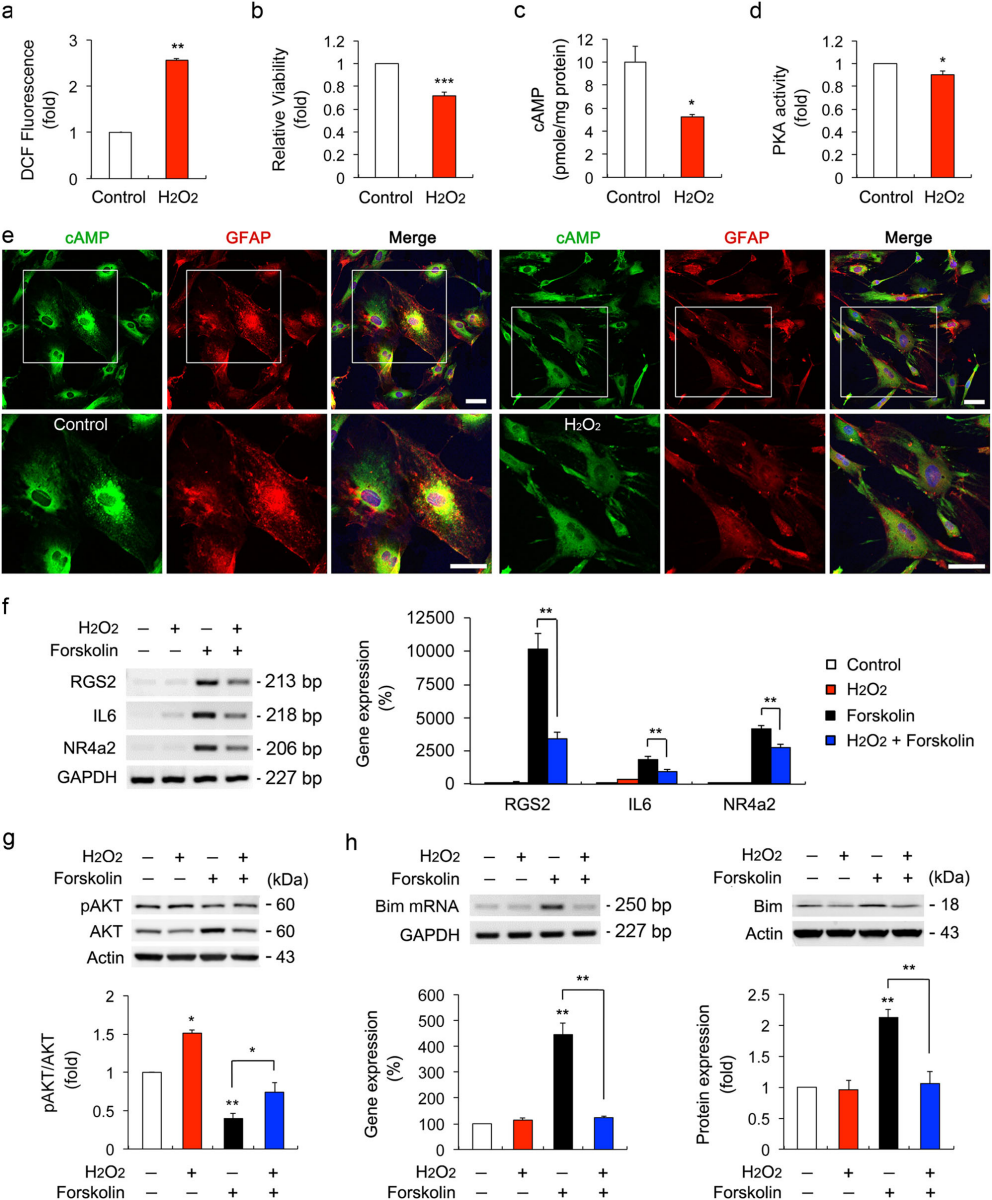
Effects of oxidative stress on cAMP/PKA signaling pathway in ONH astrocytes **a** ROS production in ONH astrocytes treated with H_2_O_2_ (50 μM) for 1 h. **b** Cell viability analysis using MTT assay in ONH astrocytes treated with H_2_O_2_ (50 μM) for 1 h. **c** Intracellular cAMP measurement in ONH astrocytes treated with H_2_O_2_ (50 μM) for 1 h. **d** PKA activity measurement in ONH astrocytes treated with H_2_O_2_ (50 μM) for 1 h. For each determination, the ROS, cell viability, PKA activity and mRNA expression in controls was normalized to a value of 1.0 and the cAMP level was normalized to the protein contents. **e** Immunocytochemical analyses of cAMP and GFAP in ONH astrocytes treated with H_2_O_2_ (50 μM) for 1 h. Representative images showed a decrease of cAMP immunoreactivity in H_2_O_2_-treated ONH astrocytes. Note that decreased level of cAMP in the cytoplasm of ONH astrocytes treated with H_2_O_2_ (50 μM). Scale bars, 20 µm. **f** Real-time RT-PCR analysis of PKA target genes in ONH astrocytes treated with H_2_O_2_ (50 μM) and/or forskolin (10 μM) for 1 h. Data were normalized by GAPDH expression. **g** Immunoblot analyses of pAKT and total AKT in ONH astrocytes treated with H_2_O_2_ (50 μM) and/or forskolin (10 μM) for 1 h. For each determination, the ratio of pAKT/total AKT protein level in control cells with no treatment was normalized to a value of 1.0. **h** Real-time RT-PCR and immunoblot analyses of Bim in ONH astrocytes treated with H_2_O_2_ (50 μM) and/or forskolin (10 μM) for 1 h. Data were normalized by GAPDH and Actin expression, respectively. For each determination, the mRNA and protein expression in controls was normalized to a value of 100% or 1.0. Data are shown as the mean ± S.D. (*n* = 3). *P < 0.05; **P < 0.01; ***P < 0.001 (two-tailed unpaired Student’s *t*-test)

However, surprisingly, oxidative stress significantly decreased the intracellular cAMP level and PKA activity in ONH astrocytes, accompanying by increased ROS production and reduced cell viability compared with the controls (Figs. 7a–d). Consistently, immunocytochemistry analysis confirmed that cAMP immunoreactivity was decreased in the cytoplasm of oxidative stress-induced ONH astrocytes (Fig. 7e). Among candidates for the PKA target genes (*nur77*, *areg*, *nr4a2*, *il-6*, *rgs2,* and *dusp1*), which are known as specific target genes of the cytosolic PKA pathway^23,24^, the expression level of *nur77* and *dusp1* mRNA was upregulated by H_2_O_2_ treatment alone but not by H_2_O_2_ and forskolin together, while *areg* mRNA expression was not changed in both H_2_O_2_- and forskolin-treated ONH astrocytes (Supplementary Fig. S5). Real-time quantitative RT-PCR analysis demonstrated that forskolin treatment alone significantly upregulated the expression levels of *rgs2, il-6,* and *nr4a2* mRNA in ONH astrocytes (Fig. 7f). However, the increased level of these genes was significantly decreased in ONH astrocytes co-treated with H_2_O_2_ and forskolin (Fig. 7f). In addition, oxidative stress significantly increased the expression level of pAKT S473 (Fig. 7g) and decreased the expression levels of Bim mRNA and protein in ONH astrocytes co-treated with forskolin (Fig. 7h). Our results also showed that there was no statistically significant difference in the expression levels of *ADCY10* (*AC10*) mRNA as well as sAC protein in cytosolic and mitochondrial fractions in ONH astrocytes treated with H_2_O_2_ (Supplementary Fig. S6).

### Inhibition of cAMP/PKA pathway protects ONH astrocytes against oxidative stress

**Fig. 8:**
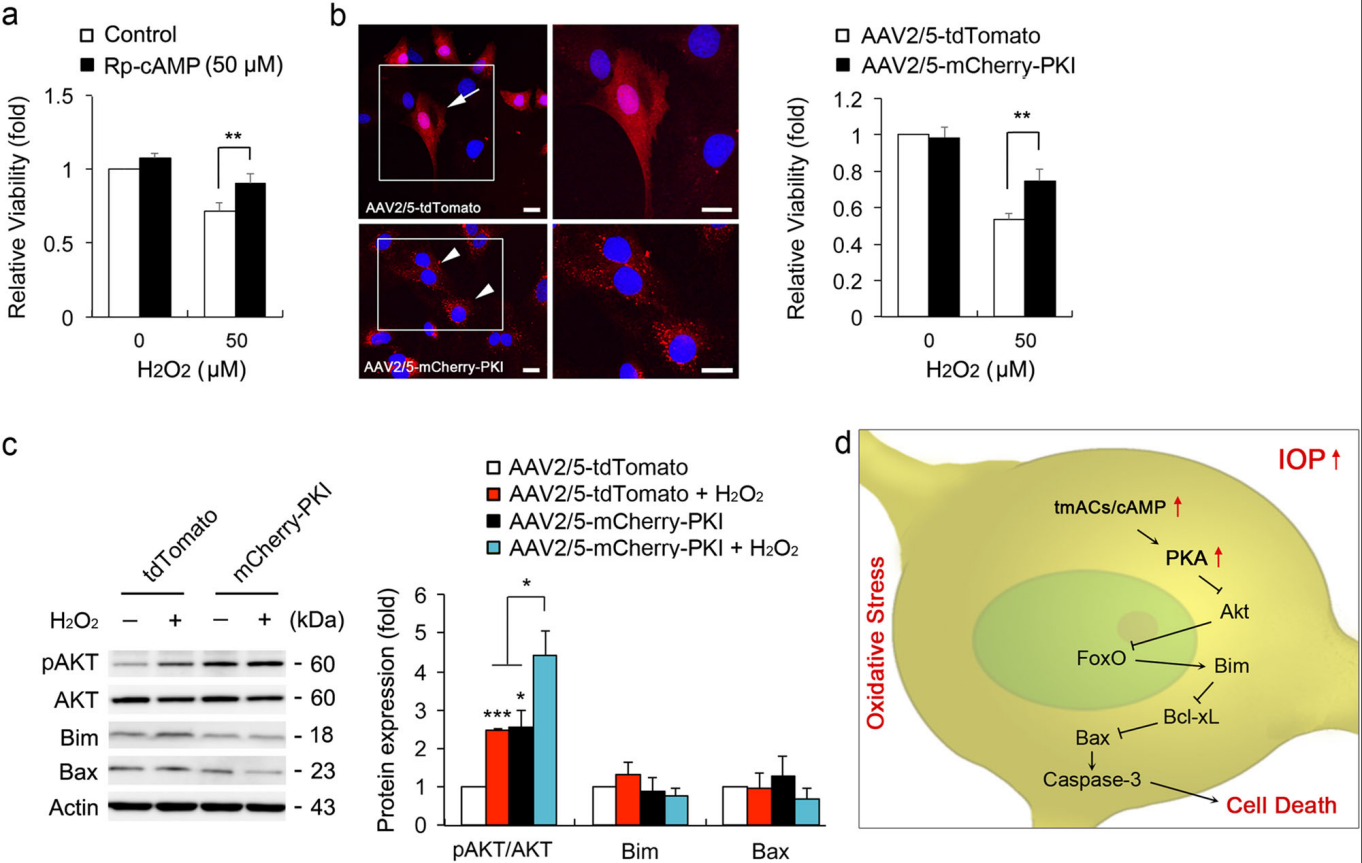
PKA inhibition protects ONH astrocytes against oxidative stress **a** Cell viability analyses using MTT assay in H_2_O_2_ (50 μM for 1 h)-induced ONH astrocytes treated with Rp-cAMP (50 μM). **b** Cell viability analysis using MTT assay in H_2_O_2_ (50 μM)-induced ONH astrocytes transduced with AAV2/5-tdTomato or mCherry-PKI. Representative images showed tdTomato (red) or mCherry-PKI (red) expression in ONH astrocytes. Nuclei (blue) were stained by Hoechst 33342. Note that PKI overexpression promoted ONH astrocyte survival. Scale bars, 20 μm. **c** Immunoblot analyses of pAKT, total AKT, Bim and activated Bax in H_2_O_2_ (50 μM for 1 h)-induced ONH astrocytes transduced with AAV2/5-tdTomato or mCherry-PKI. **d** A hypothetical model for the role of tmAC activation-mediated cAMP/PKA signaling pathway in ONH astrocytes against glaucomatous insults such as elevated IOP and oxidative stress. For each determination, the cell viability as well as mRNA and protein expression in controls were normalized to a value of 1.0. Data are shown as the mean ± S.D. (*n* = 3). *P < 0.05; **P < 0.01; ***P < 0.001 (two-tailed unpaired Student’s *t*-test)

Using Rp-cAMP or transduction of adeno-associated virus serotype 2/5 (AAV2/5)-protein kinase inhibitor (PKI) that block PKA activity, we pretreated Rp-cAMP or overexpressed PKI in ONH astrocytes. We found that PKA inhibition by Rp-cAMP or PKI overexpression significantly promoted cell viability in ONH astrocytes against oxidative stress (Figs. 8a and b). In addition, either H_2_O_2_ treatment or PKI overexpression alone significantly increased the expression level of pAKT S473 in ONH astrocytes compared with control cells (Fig. 8c). Surprisingly, the pAKT S473 was highest in PKI-expressing ONH astrocytes treated with H_2_O_2_ (Fig. 8c). However, there were no statistically significant differences in the expression levels of Bim and Bax proteins (Fig. 8c). Thus, elevated intracellular cAMP is critically involved in ONH astrocyte dysfunction via PKA activation-mediated AKT/Bim/Bax pathway, leading to caspase-3 activation and in turn, exacerbates cell death in oxidative stress (Fig. 8d).

## Discussion

In the present study, we observed that cAMP/PKA pathway inactivated AKT survival pathway in ONH astrocytes and subsequently this activated FoxO-mediated Bim/Bax death pathway, leading to caspase-3 activation-mediated cell death. Notably, elevated cAMP exacerbated the vulnerability to oxidative stress in ONH astrocytes through the PKA pathway. Another intriguing finding was that inhibition of the PKA pathway also protected ONH astrocytes against oxidative stress. We therefore propose that the inhibition of cAMP/PKA signaling pathway can be an important therapeutic target for ameliorating astrocyte dysfunction in glaucomatous ONH degeneration.

cAMP signaling pathway is critically involved in a variety of cellular function including cell survival and death^8,9,25,26^. However, little is known about the related pathophysiological mechanism and its significance on the activated cAMP signaling pathway in dysfunctional glial cells, including ONH astrocytes, in neurodegeneration. Among the main downstream effectors of cAMP signaling, PKA and Epac have a similar affinity to cAMP^27^ and contribute to cell growth and death^8^. Recent evidence showed that activation of cAMP/PKA pathway has a critical role in the apoptosis in cancer cell lines^28^ and vascular smooth muscle cells^29^. In contrast, reduced cAMP protects cultured rat cortical astrocytes via the activating AKT pathway^30–32^. Our results demonstrated that activation of cAMP/PKA pathway but not the Epac pathway diminished phosphorylation of AKT S473 in ONH astrocytes. The enhancement of cAMP/PKA activity induced caspase-3 activation-mediated cell death in ONH astrocytes. Taken together, these findings raise the possibility that inactivation of AKT by activation of cAMP/PKA pathway may be an important signal pathway in ONH astrocyte dysfunction.

AKT pathway controls the expression of apoptosis-related genes through modulating transcription factors such as NF-κB^33,34^ and FoxO^35,36^. Of note, our results showed that elevated intracellular cAMP induced a substantial increase of Bim expression, which is regulated by FoxO3a transcription factor^35,37^ in ONH astrocytes. Moreover, this upregulation of Bim expression by elevated cAMP was suppressed by PKA inhibition or AKT activation. This provides evidence that the activated cAMP/PKA pathway is linked to FoxO-mediated Bim activation in ONH astrocytes. The pro-apoptotic Bcl-2 homology domain 3-only protein Bim induces apoptosis, primarily through its increased binding activity toward multiple pro-survival Bcl-2-like proteins^38^, whose dissociations activate Bax and Bak^39^. Indeed, we demonstrated that elevated cAMP promoted a direct interaction between Bim and Bcl-xL in ONH astrocytes, and this interaction was blocked by PKA inhibition. As Bax activation leads to caspase-3 activation in the mitochondria-related intrinsic apoptosis signaling pathway^40^, longer stimulation of cAMP increased activated Bax and caspase-3 in ONH astrocytes. These findings strongly suggest the possibility that elevated cAMP contributes to cellular dysfunction through AKT inactivation and Bim/Bax activation, leading to intrinsic apoptosis signaling pathway, in ONH astrocytes.

We demonstrated that PKA inhibition by Rp-cAMP significantly inhibited activation of both Bax and caspase-3, whereas Rp-cAMP could not restore AKT phosphorylation in forskolin-treated ONH astrocytes. Interestingly, however, PKA inhibition by H89 restored AKT phosphorylation, whereas H89 could not prevent activation of both Bax and caspase-3 activation in forskolin-treated ONH astrocytes. It is possible that prolonged activation of PKA might not completely be prevented by Rp-cAMP because it is a competitive inhibitor of the cAMP-binding site of PKA regulatory subunits and its effect is likely to be diminished when endogenous levels of cAMP are extremely high^41^. This notion is also supported by the effect of PKI overexpression on pAKT expression under prolonged PKA activation by forskolin and rolipram treatment for 24 h. Although pAKT expression was increased in PKI-overexpressing ONH astrocytes compared with control cells, PKI did not restore the reduction of pAKT expression. However, interestingly, PKI significantly decreased Bax activation (Supplementary Fig. S7), showing a similar pattern of the effect of Rp-cAMP on PKA. As PKI specifically inhibits PKA activity by binding to the free catalytic subunit, which mimics the function of regulatory subunit of PKA^42^, it is also not sufficient to block the prolonged activation of PKA completely like as Rp-cAMP. However, H89 inhibits catalytic subunits of PKA directly to competing with ATP and it could block completely the prolonged activation of PKA more efficiently than Rp-cAMP and PKI^43^. Furthermore, H89 also inhibits nonspecifically other kinases including Rho-associated kinase in a PKA-independent manner^41^. Therefore, the pharmacological drugs that modulate the regulatory subunit of PKA may be more attractive than the drugs that modulate catalytic subunit activity for promoting preventive effect.

As increased cAMP is associated with the promotion of neurotrophic sensitivity in RGC survival^44,45^, recent evidence suggested that RGC survival and axon growth are enhanced by the activation of sAC-generated cAMP^9,25^ but not likely to be tmACs dependent by examining the effects of tmACs inhibitors or the AC1/AC8 double knockout mice^25^. These results suggested that sAC-generated cAMP has a critical role for RGC survival and axon growth, whereas tmACs-generated cAMP has a minor effect on RGC survival and axon growth. Surprisingly, however, our results demonstrated that elevated cAMP by forskolin treatment showed a beneficial effect by promoting cell survival in RGCs. In contrast, pharmacological inhibition of cAMP/PKA activation lowered the cell viability in RGCs. Based on these results demonstrated here, it is possible that elevated cAMP has distinctive roles in the cellular processes of RGC axons and astrocytes in glaucomatous ONH degeneration. Previous studies have reported that glaucomatous damage induced increase of two tmACs, *AC3* and *AC9*, gene expression in the unstimulated ONH astrocytes from AA donors with POAG as well as in the elevated hydrostatic pressure-induced ONH astrocytes from normal AA donors^11,12^. Consistently, these results were supported by the observation of increased expression levels of cAMP, Bax and caspase-3 protein in the astrocytes of the glial lamina in glaucomatous DBA/2 J mice, accompanied by structural loss of astrocytic processes and degenerative axons. Together, these results suggest another possibility that elevated cAMP-dependent pathway is an important player in not only ONH astrocytes but also RGC axons against glaucomatous insults such as elevated IOP and oxidative stress. Collectively, our results identify for the first time a pathological link between cAMP/PKA pathway activation and AKT/Bim/Bax-mediated intrinsic cell death pathway in ONH astrocyte dysfunction.

Oxidative stress is considered as a key risk factor in many neurodegenerative diseases including glaucoma^2,4,13,15^. Our results show that elevated cAMP exacerbated the vulnerability of ONH astrocytes against oxidative stress. As a well-known molecular biomarker in POAG^46,47^, an increase of TNFα expression has been reported in glial cells of the ONH from patients with glaucoma^48–50^, and glial cell-derived TNFα signaling pathway is associated with oxidative stress and RGC axon degeneration in glaucomatous ONH^48,50,51^. In the present study, our results demonstrated that elevated intracellular cAMP may not only activate TNFα by inducing the splicing of TNFα mRNA in ONH astrocytes but also accelerated TNFα activation by enhancing the splicing of TNFα mRNA in ONH astrocyte in synergy with oxidative stress. On the other hand, intriguingly, our results also demonstrated that oxidative stress alone induced the reduction of cAMP level and PKA activity in ONH astrocytes. Therefore, our findings support the notion that elevated cAMP-mediated enhancement of TNFα activation could be critical for oxidative stress-mediated ONH astrocyte dysfunction and RGC axon degeneration in POAG.

On the other hand, intriguingly, our results also demonstrated that oxidative stress alone decreased cAMP level and PKA activity and subsequently activated AKT pathway in ONH astrocytes. How is this possible? In the central nervous system, neurons are most vulnerable cells to oxidative stress due to their low ROS- detoxifying capacity, therefore its survival is highly dependent on the capacity of neighboring astrocytes during oxidative stress-induced neurodegeneration^52,53^. Emerging evidence suggests that AKT activity is critical for astrocyte resilience to oxidative stress and enhances the protective effect of astrocytes against oxidative stress^54^. Indeed, we observed that oxidative stress activated AKT pathway in ONH astrocytes, accompanied by restoration of Bim expression. Because a rapid and transient increase in relatively lower concentration of H_2_O_2_ (25 *µ*M) promotes the pro-survival activity in photoreceptor cells against serum deprivation by increasing AKT phosphorylation^55^, our results raise a possibility that a transient induction of oxidative stress (relatively low concentration of H_2_O_2_) in ONH astrocytes may trigger the reduction of cAMP level and PKA activity by a rapid and transient activation of AKT survival pathway, which in turn, it may inhibit Bim activation. In addition, our results also demonstrated that enhanced AKT phosphorylation by IGF-1 rescued ONH astrocytes against elevated cAMP. These results suggest that increasing AKT phosphorylation may be an important endogenous compensatory defense mechanism against oxidative stress, which is mediated by inactivation of cAMP/PKA signaling pathway, in ONH astrocytes. Therefore, the dysregulation of the defense system by elevated cAMP level may accelerate ONH astrocyte dysfunction in oxidative stress.

Recent evidence has demonstrated that inhibition of PKA pathway by H89 protected hippocampus against Aβ-induced oxidative stress via increases of glutathione levels and superoxide dismutase activity^56^. Furthermore, anti-apoptotic markers, such as Bcl-xL, pBad, and heat shock protein 70, are increased in the brain of aged AC5 knockout mouse that shows resistance against oxidative stress^57^. Our results indicated that PKA inhibition by Rp-cAMP treatment promoted cell viability in ONH astrocytes against oxidative stress. Furthermore, PKI overexpression protected ONH astrocytes against oxidative stress by enhancing AKT phosphorylation. Taken together, these results further support the notion that activation of cAMP/PKA pathway may exacerbate vulnerability to oxidative stress in ONH astrocytes and consequently may promote susceptibility to the factors contributing to progressive axon degeneration in POAG.

In summary, we provide evidence that the cAMP/PKA signaling pathway is important to the vulnerability of oxidative stress-induced ONH astrocytes and that inhibition of intracellular cAMP/PKA signaling activation may protect ONH astrocytes by increasing AKT phosphorylation against oxidative stress. These results suggest that modulating cAMP/PKA signaling pathway has therapeutic potential for glaucoma pathogenesis.

## Methods

### Animals

Pregnant Sprague–Dawley rats (250–300 g in weight; Harlan Laboratories) and adult female DBA/2 J and DBA/2J-*Gpnmb*^*+*^ mice (The Jackson Laboratory) were housed in covered cages, fed with a standard rodent diet *ad libitum*, and kept on a 12 h light/12 h dark cycle. All procedures concerning animals were in accordance with the Association for Research in Vision and Ophthalmology Statement for the Use of Animals in Ophthalmic Vision Research and under protocols approved by Institutional Animal Care and Use Committee at the University of California, San Diego.

### Reagents and other materials

Chemicals and other materials were obtained from the following sources: H_2_O_2_, forskolin, Rp-cAMP, and H89 from Sigma; Rolipram from Cayman Chemical; Recombinant Rat IGF-1 from Prospec; DMEM/F12 (1:1), MEM+GlutaMax, 100 × penicillin–streptomycin (Pen/Strep) and fetal bovine serum from Gibco; Superscript II reverse transcriptase from Invitrogen; RNase inhibitor, dNTP mixture and oligo (dT) from Promega; PCR master mix, protein size marker, SuperSignal West Pico and Femto chemiluminescent substrates from Thermo scientific; Amersham Hybond-P (PVDF membrane) and ECL Prime Western Blotting Detection Reagent from GE Healthcare; Lab-Tek Chambered cover glass from Nunc; CE3F4 from TOCRIS.

### Tissue preparation

Mice were anesthetized with intraperitoneal injection of a mixture of ketamine (100 mg/kg, Ketaset; Fort Dodge Animal Health) and xylazine (9 mg/kg, TranquiVed; VEDCO Inc.) before cervical dislocation. For immunohistochemistry, the retinas and ONHs were dissected from the choroids and fixed for 2 h at 4 °C with 4% paraformaldehyde (Sigma) in phosphate-buffered saline (PBS, pH 7.4). After several washes in PBS, the retinas were dehydrated through graded ethanols and embedded in polyester wax. For immunoblot analyses, extracted retinas were immediately used.

### Primary ONH astrocyte culture

Primary rat ONH astrocytes were prepared as described previously with minor modifications^16^. In brief, after euthanizing with CO_2_ gas, 10–20 pieces of ONH tissue were dissected from postnatal day 5 Sprague–Dawley rats and transferred to a 35-mm petri dish with 2 ml 0.2% bovine serum albumin (BSA)/Dulbecco’s phosphate-buffered saline (DPBS). Under a dissecting microscope, remnant tissues were removed, and the ONH tissue was identified and dissected using a micro-scissors and sharp blade. The ONH tissues were minced and transferred to a 60-mm petri dish and conditioned with growth medium: MEM+GlutaMax (Gibco) supplemented with 10% fetal bovine serum, 100 U/ml penicillin and 100 μg/ml streptomycin (Gibco), and incubated at 5% CO_2_ at 37 °C. After incubation for 1–2 weeks, the ONH explants were removed by 70 μm cell strainers (BD Biosciences). The cells that were grown from the ONH explants were plated in a 100 mm culture dish and cultured until 80% confluence. For further purification of the ONH astrocytes, the culture dish was shaken for 16 h at room temperature, followed by growth medium changing with serum-free medium and the cells were incubated for 24 h at 5% CO_2_ at 37 °C. After removing non-adherent cells, the adherent ONH astrocytes were collected, centrifuged and plated on poly-l-lysine (PLL)-coated culture dishes.

### Primary RGC culture system

RGCs from postnatal 3–5 days of Sprague–Dawley rats were purified by immunopanning as described previously^58,59^. In brief, ~15,000 purified cells were seeded on 24-well plates coated first with poly-d-lysine (10 μg/ml; Sigma) and then with laminin (10 μg/ml; Sigma) in neurobasal medium. RGCs were cultured in serum-free defined growth medium containing BDNF (50 μg/ml; PeproTech), CNTF (10 μg/ml; Sigma), insulin (5 μg/ml; Sigma), and forskolin (10 μM; Sigma).

### Cell viability assay

Cell viability was measured using MTT according to the manufacturer’s recommendations (Cell Proliferation Kit1; Roche Diagnostics). In brief, ONH astrocytes were plated on a 96-well plate (0.5~1 × 10^4^ per well). After 48 h, the cells were treated with various reagents for 1 h and 10 μl MTT stock solution was added to each well including the negative control. The cells were incubated for 4 h in a humidified atmosphere of a 5% CO_2_ incubator at 37 °C and 100 μl of solubilization solution were added to dissolve the formazan crystals that remained in the wells. After incubation for overnight at 5% CO_2_ at 37 °C, the absorbance at 560 and 690 nm were measured with a microplate reader (SpectraMAX; Molecular Devices). Each set of data was collected from multiple replicate wells of each experimental group (*n* = 3).

### Cell death assay

Dead and membrane-ruptured cells were quantitatively measured by LDH release assay. LDH activity in cultured medium was assessed with Cytotoxicity Detection Kit (LDH; Sigma) according to the manufacturer’s instructions. In brief, After ONH astrocytes were treated with forskolin (10 µM) and/or H_2_O_2_ (50 µM) for 24 h, 100 µl of cell culture medium of each group were mixed with the 100 µl reaction mixture in a microplate followed by 30 min incubation at room temperature in dark environment. The absorbance of the samples was measured at 490 nm using a microplate leader (SpectraMAX, Molecular Devices). Two percent triton X-100-treated samples were used as positive controls. Each set of data was collected from multiple replicate dishes of each experimental group (*n* = 3).

### RT-PCR and quantitative real-time RT-PCR

In brief, total RNA was isolated from the cells using the RNeasy mini kit (Qiagen), according to the manufacturer’s protocol. cDNAs were synthesized from total RNAs with Superscript II reverse transcriptase and oligo (dT) primers according to the manufacturer’s protocols. RT-PCR was performed with cDNAs synthesized from 0.1 μg of the total RNA of each cell as a template and specific primers (Supplementary Table 1). RT-PCR products were electrophoresed on a 2% agarose gel and visualized by ethidium bromide staining. For the quantification of the relative mRNA expressions of each group, real-time PCR was carried out using MX3000P real-time PCR system (Stratagene) as follows. cDNAs were amplified using iQ SYBR Green super-mix (Bio-Rad) and the specific primers for 40 cycles (initial incubation at 50 °C for 2 min and then at 95 °C for 10 min, and 40 cycles (95 °C for 15 sec, 55 °C for 1 min, and 72 °C for 1 min)). Output data were obtained as *Ct* values and the differential mRNA expression of each gene among samples was calculated using the comparative *Ct* method. GAPDH mRNA, an internal control, was amplified along with the target genes, and the *Ct* value of GAPDH was used to normalize the expression of target genes.

### Immunohistochemical and immunocytochemical analyses

For immunohistochemistry of tissue section, five sections (7 μm thickness) per wax block of ONHs from 10-month-old glaucomatous DBA/2 J and age-matched non-glaucomatous DBA/2J-*Gpnmb*^*+*^ mice (*n* = 3 mice/group) were prepared. Following incubation in 1% BSA/PBS for 1 h at room temperature to prevent nonspecific background, the sections were incubated with the primary antibodies for 16 h at 4 °C. The primary antibodies were mouse monoclonal anti-cAMP antibody (1:500; abcam), guinea pig polyclonal anti-GFAP antibody (1:500; Advanced Immuno Chemical, Inc.), and rabbit polyclonal caspase-3 (1:100; Cell signaling). After several washing, the sections were incubated with the secondary antibodies Alexa Fluor 488 dye-conjugated goat anti-mouse IgG (1:100; Invitrogen) and Cy5-conjugated anti-guinea pig IgG antibody (1:100; Jackson ImmunoResearch Laboratories) for 2 h at 4 °C and were subsequently washed with PBS. The sections were counterstained with Hoechst 33342 (1 μg/ml; Invitrogen) in PBS. For immunocytochemistry of cultured ONH astrocytes, the cells were fixed with 4% paraformaldehyde/PBS for overnight at 4 °C. After blocking nonspecific background with 1% BSA/PBS for 1 h at room temperature, primary antibodies were incubated for 16 h at 4 °C. The primary antibodies were mouse monoclonal anti-cAMP antibody (1:500; Sigma), mouse monoclonal anti-Bax antibody (6A7; 1:100; Santa Cruz), and rabbit polyclonal caspase-3 (1:100; Cell signaling). After several wash steps, the cells were incubated with the secondary antibody, Alexa Fluor 488 dye-conjugated goat anti-mouse IgG antibody (1:100; Invitrogen) or Alexa Fluor 488 dye-conjugated goat anti-rabbit IgG antibody (1:100; Invitrogen) for 4 h at 4 °C and subsequently washed with PBS. The cells were counterstained with Hoechst 33342 (1 μg/ml, Life Technologies) in PBS. Images were acquired with confocal microscopy (Olympus FluoView1000; Olympus). ImageJ (http://rsb.info.nih.gov/ij/) was used to measure the fluorescence intensity in pixels per area in each cAMP, GFAP, Bax, and caspase-3 images from glaucomatous DBA/2 J and age-matched control DBA/2J-*Gpnmb*^*+*^ mice. In each antibody image acquisition, all imaging parameters remain the same. Mean pixel intensity was measured in this 11,124 square pixel area.

### cAMP measurement

Cells (2~5 × 10^5^) were washed in ice-cold PBS, harvested by centrifugation at 1800 g for 5 min and extracted with 0.1 M HCl. The extracts were centrifuged at 15,000 g for 3 min and the supernatants were collected. Intracellular cAMP levels in the final supernatants were measured by using a direct cAMP ELISA kit (Enzo Life Sciences) according to the manufacturer’s instructions for acetylation format. Protein concentration of the same samples was determined by Bradford protein quantification assay. Data were normalized to the protein amounts.

### ROS measurement

The intracellular ROS was measured by using cell permeant reagent 2’,7’–dichlorofluorescin diacetate (DCFHDA, Sigma). In brief, ONH astrocytes were plated on a PLL-coated six-well plate (2 × 10^4^ cell per well) and after 24 h, cells were exposed to 50 µM of H_2_O_2_ for 3 h in serum-free condition. The cells were added with 200 nM of DCFHDA, incubated at 37 °C for 20 min and then detached with 0.25% typsin/EDTA. After washing with DPBS, the fluorescence intensity of the sample was measured immediately using C6 Accuri flow cytometer (BD Biosciences). Each set of data was collected from multiple replicate dishes of each experimental group (*n* = 3).

### PKA activity assay

PKA activity in the samples from ONH atrocytes was measured by using a non-radioactive PKA activity assay kit (Enzo Life Sciences). In brief, ONH astrocytes (2 × 10^5^) were seeded on PLL-coated 200 mm dishes in normal growth medium for 48 h. The cells were treated with indicated reagents in serum-free condition for 1 h. Total cellular proteins were extracted by using lysis buffer according to the manufacturer’s instruction. PKA substrate-coated microtiter plate was soaked with kinase assay dilution buffer for 10 min at room temperature. One hundred nano-gram in 30 µl of cell lysates or PKA standard (10 ng) were then added, followed by the addition of ATP to initiate the kinase reaction. After incubation at 30 °C for 90 min, the reaction was terminated by removing the mixture from the plate, and phosphor specific substrate antibody was added to each well and incubated at room temperature for 60 min. The solution was removed completely and the wells were repeatedly washed. The peroxidase-conjugated secondary anti-rabbit IgG was then added to each well and incubated for another 30 min at room temperature. After several washing, the color was developed with tetramethylbenzidine substrate for 30–60 min and the reaction was stopped by adding acid-stop solution. The absorbance was measured at 450 nm with a microplate reader (SpectraMAX; Molecular Devices). The absorbance was divided by the concentration of total protein (µg) in each sample, and the data are represented as relative PKA activity.

### Co-IP and immunoblot analyses

For co-IP, the cell lysates were prepared in co-IP lysis buffer (20 mM Tris-Cl, pH 7.4, 135 mM NaCl, 1.5 mM MgCl_2_, 1 mM EGTA, 10% glycerol, 1% Triton X-100) containing complete protease inhibitors (Roche Biochemicals). IP was performed using Protein A/G PLUS-Agarose Immunoprecipitation Reagent (Santa Cruz Biotechnology) and a rabbit polyclonal antibody to Bim (Santa Cruz Biotechnology) according to the manufacturer’s instructions. For immunoblot analyses, cells were harvested and lysed for 30 min on ice with a modified RIPA lysis buffer (150 mM NaCl, 1 mM EDTA, 1% NP-40, 0.1% SDS, 1 mM DTT, 0.5% sodium deoxycholate, and 50 mM Tris-Cl, pH 7.6), containing the complete protease inhibitors. The lysates were centrifuged at 15,000 g for 15 min and the protein amounts in the supernatants were measured by Bradford methods. Proteins (5–10 μg) were separated by SDS/PAGE and electrotransferred to polyvinylidenedifluoride membrane. The membrane was blocked with 5% non-fat dry milk and PBS-T (0.1% Tween-20) for 1 h, incubated with primary antibodies for overnight at 4 °C. Primary antibodies are rabbit polyclonal anti-AKT antibody (1:5000; Cell Signaling), mouse monoclonal anti-pAKT antibody (S473, 1:2000; Cell Signaling), rabbit polyclonal anti-pFoxO1/3 (T24,T32, 1:1,000; cell signaling), rabbit polyclonal anti-caspase-3 antibody (1:3000; Cell Signaling), mouse monoclonal anti-Bax antibody (6A7; 1:1000; Santa Cruz Biotechnology), rabbit polyclonal anti-Bcl-xL antibody (1:2000; Cell Signaling), mouse monoclonal anti-Bcl-xL antibody (1:1000; Santa Cruz Biotechnology), rabbit polyclonal anti-Bim antibody (1:1000; Santa Cruz Biotechnology), and mouse monoclonal anti-Actin antibody (1:100,000; Millipore), After several washes in PBS-T, the membranes were incubated with peroxidase-conjugated goat anti-mouse or rabbit IgG (1:5000; Bio-Rad), and developed using enhanced chemiluminescence substrate system. The images were captured and quantified by using ImageQuant LAS 4000 system (GE Healthcare Bio-Science) and the band densities were normalized to the band densities for actin.

### SBEM

ONH tissues were washed with buffer and then placed into 2% OsO_4_/1.5% potassium ferrocyanide in either 0.15 M CB containing 2 mM CaCl_2_. The tissues were left for 2 h at room temperature. After thorough washing in double distilled water, the tissues were placed into 0.05% thiocarbohydrazide for 30 min. The slices were again washed and then stained with 2% aqueous OsO_4_ for 1 h. The tissues were washed and then placed into 2% aqueous uranyl acetate overnight at 4 °C. The tissues were washed with water at room temperature and then stained with en bloc lead aspartate for 30 min at 60 °C. The tissues were washed with water and then dehydrated on ice in 50, 70, 90, 100, 100% ethanol solutions for 10 min at each step. The tissues were then washed twice in dry acetone and then placed into 50:50 Durcupan ACM:acetone overnight. The tissues were transferred to 100% Durcupan resin overnight. The tissues were then embedded and left in an oven at 60 °C for 72 h. SBEM data were collected with a 3View unit (Gatan Inc.) installed on a Merlin field emission SEM (Carl Zeiss Microscopy). The ONH volumes were collected in 2.0–2.4 kV accelerating voltages, with a raster size of 24k × 24k and pixel dwell time of 0.5–1 μs. The pixel sizes were 4.0–7.3 nm, depending on the raster size and section thickness was 60–70 nm. Once a volume was collected, the histograms for the tissues throughout the volume stack were normalized to correct for drift in image intensity during acquisition. Digital micrograph files (.dm4) were normalized using Digital Micrograph and then converted to MRC format. The stacks were converted to eight bit and volumes were manually traced for reconstruction and analysis using IMOD software (http://bio3d.colorado.edu/imod/)^60^.

### *In vitro* transduction of recombinant AAV2/5 constructs

Recombinant AAV2/5 was prepared by using AAV Helper-Free System (Agilent Technology) with some modification. To generate an AAV2/5 capable of expressing mCherry-PKI fusion protein in astrocytes, AAV2/5 rep, and cap plasmid was purchased from Penn Vector Core (University of Pennsylvania) and pZac2.1 gfaABC1D-tdTomato^61^ was a gift from Baljit Khakh (Addgene plasmid # 44332). To construct pZac2.1 gfaABC1D-mCherry–PKI, the cDNA for mCherry-PKI was amplified from p4mt-mCherry-PKI that was kindly provided by Dr. Giulietta Di Benedetto^62^ by using following primers; Forward 5’-AAGCTTGAATTCGCCACCATGGTGAGCAAGGGCGAGGAGGA-3’ and reverse 5’-AAGCTTGCGGCCGCCTATGACTCGGACTTAGCAG-3’. The tdTomato portion of pZac2.1 gfaABC1D-tdTomato was replaced with mCherry-PKI at *EcoR* I and *Not* I site. After DNA sequence validation, these plasmids were used for AAV2/5 production. Viruses were prepared according to manufacturer’s instruction (AAV Helper-Free System; Agilent Technology) and purified by a column chromatography using a ViraTrap AAV purification kit (Biomiga) as described in the manufacturer’s protocol. Viral titers were determined using a quantitative real-time PCR method. The both AAV2/5 titers were 2.0 × 10^12^ GC/ml and transduction was performed to ONH astrocytes with 400,000 MOI.

### Statistical analyses

Data were presented as the mean ± S.D. Comparison experimental conditions was evaluated using the two-tailed unpaired Student’s *t*-test with Bonferroni correction. *p* < 0.05 was considered to be statistically significant.

## Electronic supplementary material

Supplementary Movie S1

Supplementary Movie S2

Supplementary Movie S3

Supplementary Information
