# Supplementary material for: Elevated intracellular cAMP exacerbates vulnerability to oxidative stress in optic nerve head astrocytes
Source: Cell Death Dis. 2018 Feb 19;9(3):285. doi: 10.1038/s41419-017-0171-8 (PMC5833440; doi:10.1038/s41419-017-0171-8)
Supplement: Supplementary file 4 — Supplementary Information [file 41419_2017_171_MOESM4_ESM.pdf]

Supplementary Information

Elevated intracellular cAMP exacerbates vulnerability to oxidative stress in optic nerve head astrocytes

Myoung Sup Shim[1], Keun-Young Kim[2], Jung Hyun Bu[1], Hye Seung Nam[1], Seung Won Jeong[1], Tae Lim Park[1], Mark H. Ellisman[2], Robert N. Weinreb[1], Won-Kyu Ju[1]

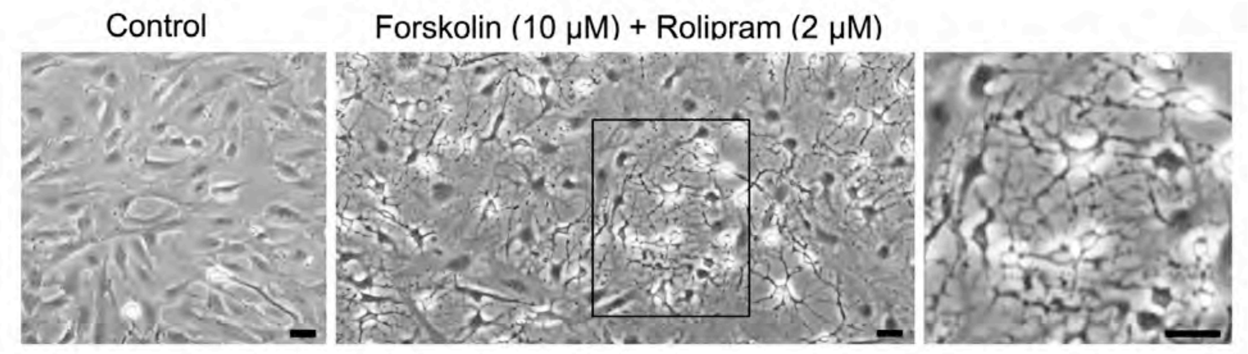

Supplementary Figure S1. Morphological change in ONH astrocytes treated with forskolin and rolipram. The cells were treated with forskolin and rolipram and examined under an inverted microscope and photographed after 24 h treatment. Each panel shows the morphology of the cells. Magnified images are shown in inboxes.

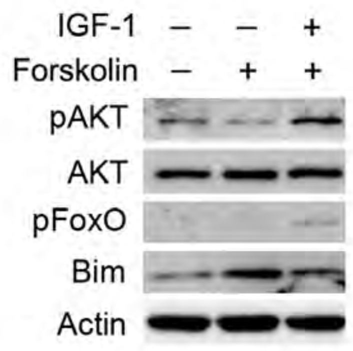

Supplementary Figure S2. Increased expression of pAKT and pFoxO protein, but decreased expression of Bim protein in cultured ONH astrocytes. Immunoblot analyses of pAKT, AKT, pFoxO and Bim in ONH astrocytes treated with forskolin (10 μM) or forskolin plus IGF-1 (100 nM) for 24 h. Full-length blots are presented in Supplementary Figure S13.

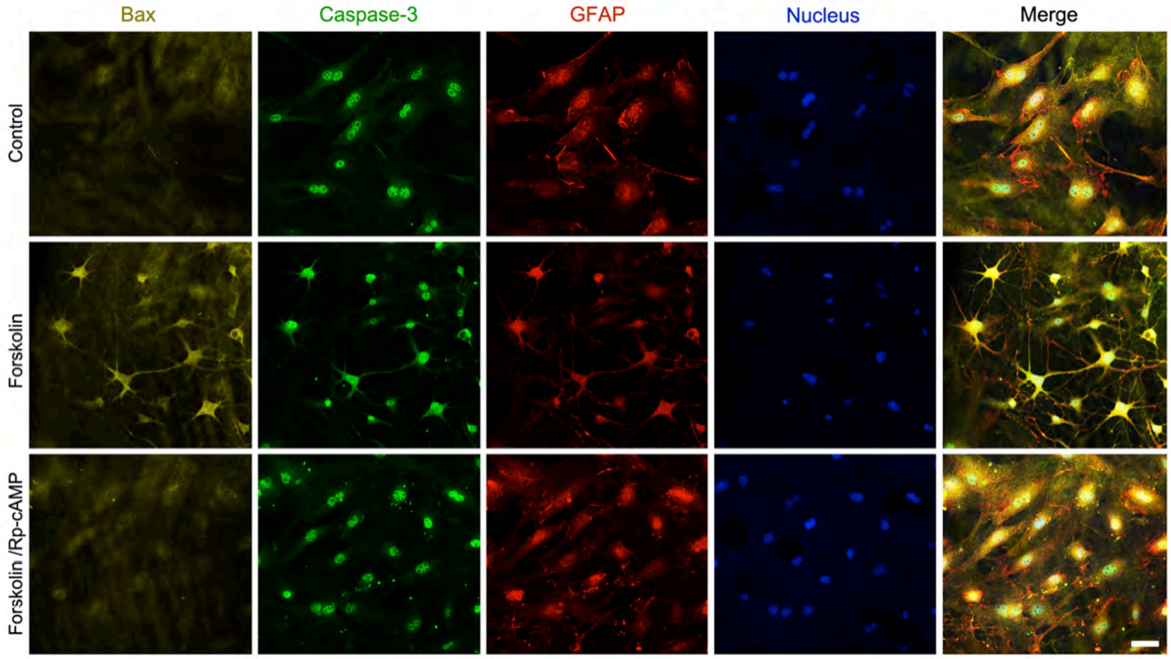

Supplementary Figure S3. Increased expression of active Bax and caspase-3 protein in cultured ONH astrocytes. Immunocytochemical analysis of active Bax, caspase3 and GFAP immunoreactivity in ONH astrocyte treated with forskolin (10 μM) or forskolin (10 μM) plus Rp-cAMP (50 μM) for 1 h. Scale bar, 20 μm.

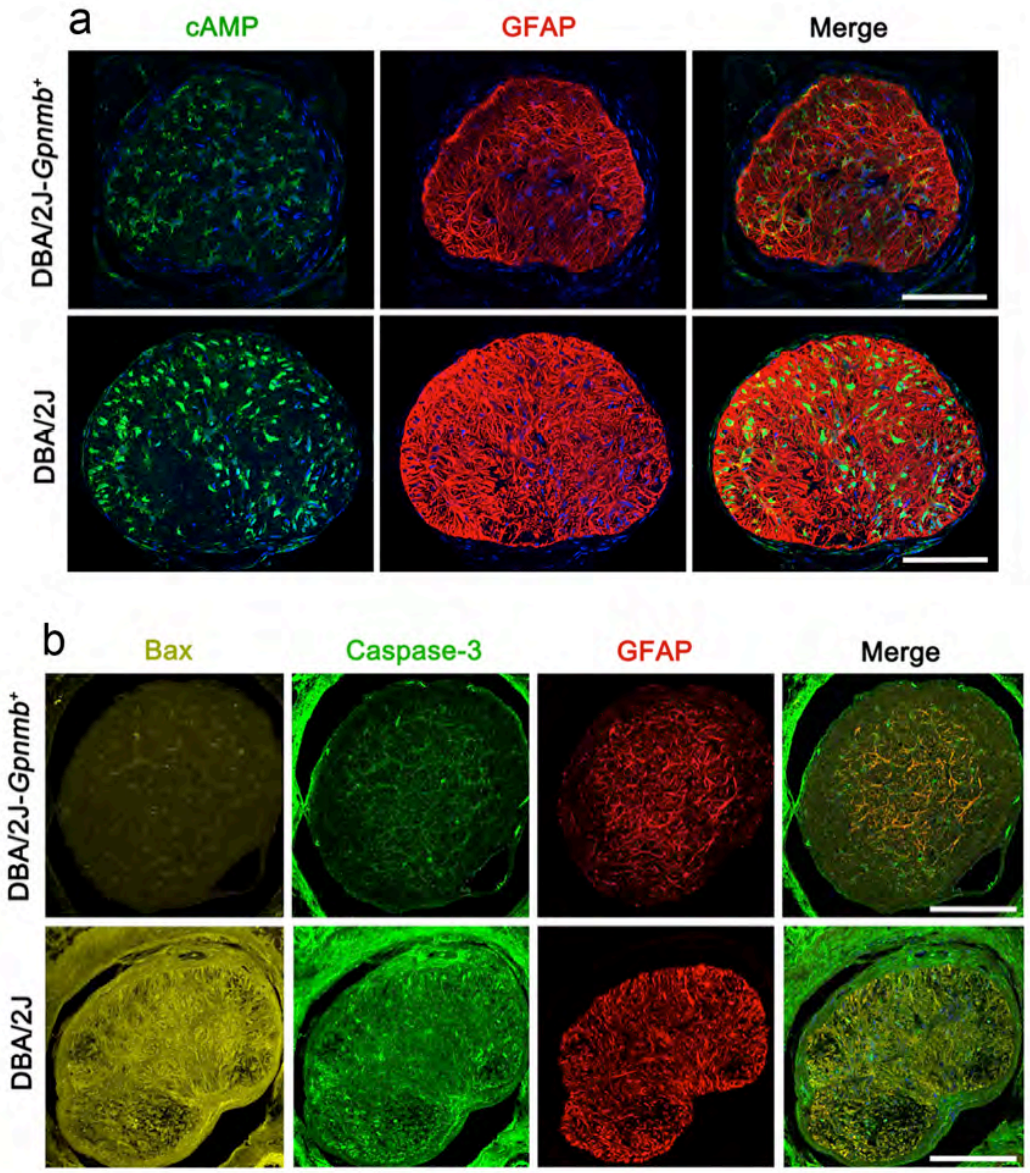

Supplementary Figure S4. Increased cAMP as well as active Bax and caspase-3 in astrocytes of the glial lamina in glaucomatous DBA2/J mice. Note that representative images showed increases of cAMP, Bax and caspase-3 immunoreactivity in astrocytes of the glial lamina in glaucomatous DBA/2J mice compared with non-glaucomatous DBA/2J-*Gpnmb*$^{+}$. Nuclei were stained with Hoechst 33342. Scale bar, 50 μm.

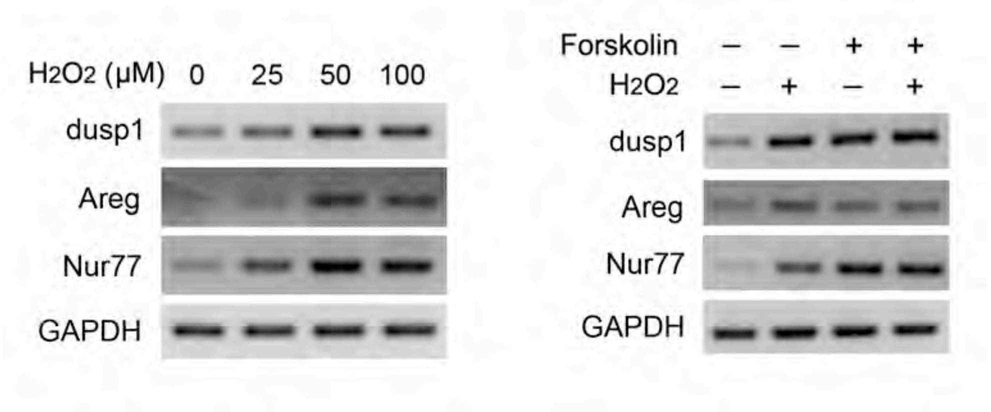

Supplementary Figure S5. RT-PCR analysis for mRNA expression level of dusp1, Areg and Nur77 in H_2O_2 and/or forskolin-treated ONH astrocytes. The mRNA level of genes indicated was measured by RT-PCR and representative images from agarose gel electrophoresis of RT-PCR analyses were shown.

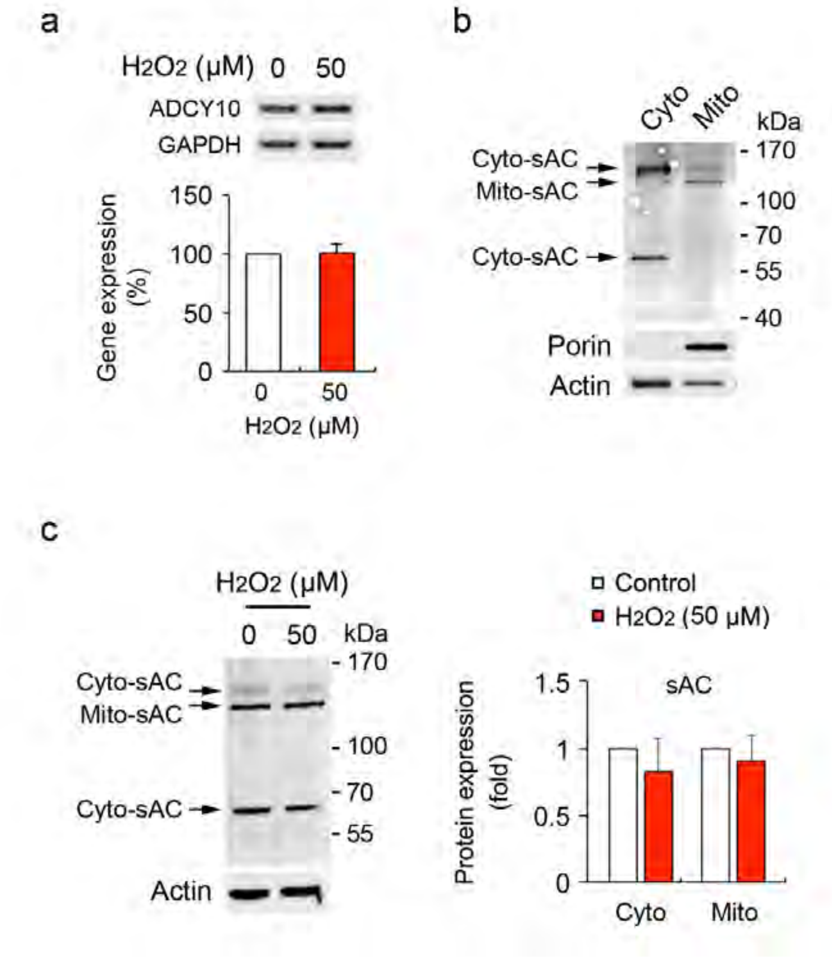

Supplementary Figure S6. Expression of ACDY10 *gene* and sAC protein in oxidative stress-induced ONH astrocytes. (a) Real-time RT-PCR analysis of *ACDY10* gene in ONH astrocytes treated with H_2O_2 (50 µM) for 1 h. For each determination, the gene expression in controls was normalized to a value of 1.0. **(b)** Immunoblot analyses of cytosolic and mitochondrial sACs in ONH astrocytes treated with H_2O_2 (50 µM) for 1 h. For each determination, the mRNA and protein expression in controls was normalized to a value of 100% or 1.0. Data are shown as the mean ± S.D (n = 3).

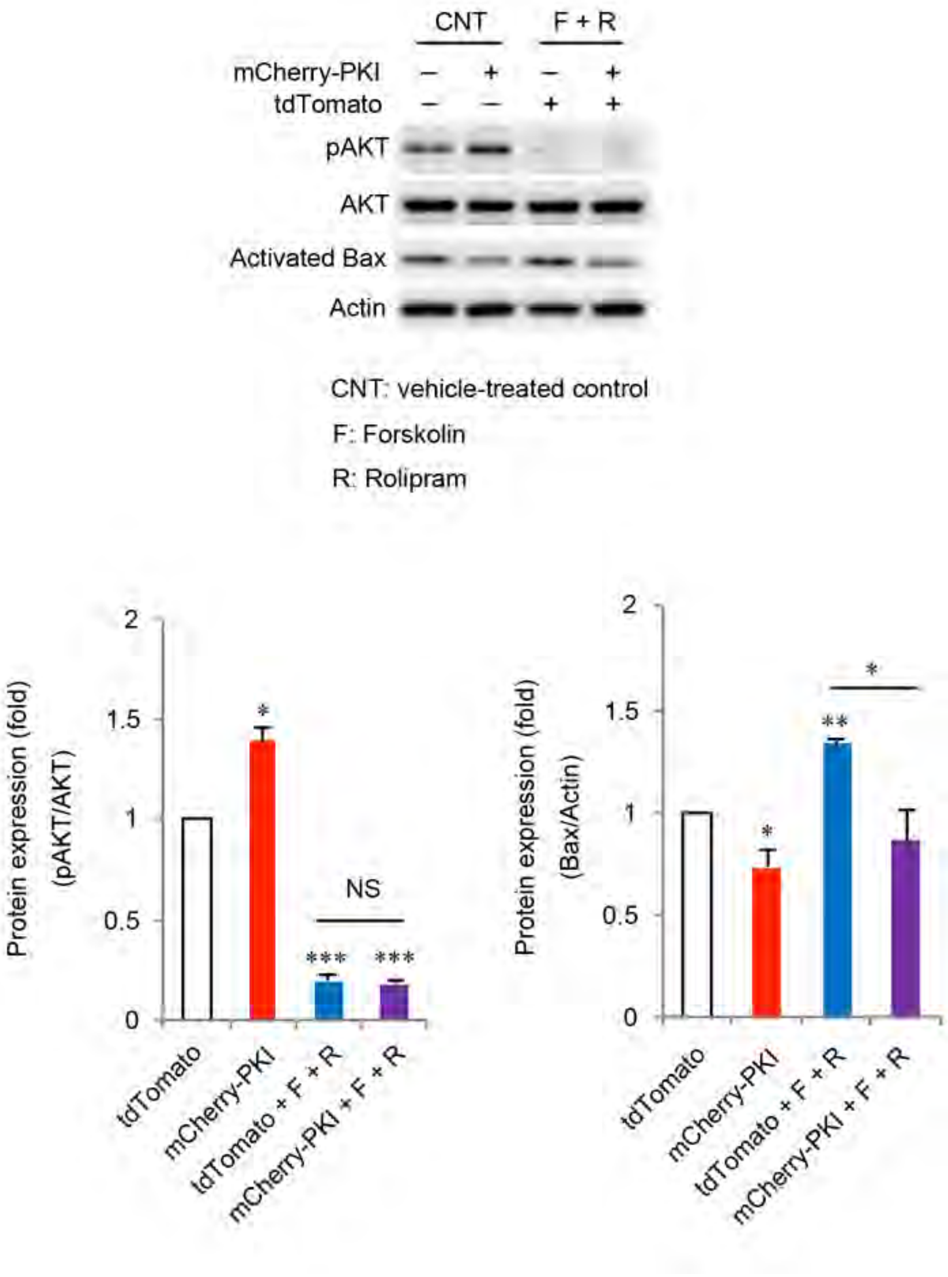

Supplementary Figure S7. The effect of mCherry-PKI overexpression on pAkt and activated Bax expression in ONH astrocytes. Immunoblot analyses of pAKT, AKT and activated Bax in mCherry-PKI-overexpressing ONH astrocytes in the absence or presence of forskolin (10 μM) and rolipram (2 μM) for 24 h. For each determination, the ratio of pAKT/AKT protein and activated Bax expression in controls was normalized to a value of 1.0. Data are shown as the mean ± S.D (n = 3). *$P < 0.05$; **$P < 0.01$; ***$P < 0.001$ (two-tailed unpaired Student's t-test).

Supplementary Table S1

Primers used in the study

	Forward Primer	Reverse primer
Adcy10	aagggcaagtcctccatctt	cagcaaattcccctgtgtct
Areg	cggaaaaggcagaagaaaca	cttacggcggagacaaagac
Bcl2	cttcagggatggggtgaact	atcaaacagaggtcgcatgc
Bcl6	acaagacggtccacacaggt	ggtacggcttctctccagtg
bclxL	agcgtagacaaggagatgca	tcaacaaccatgccaggaga
Bim	caacacaaaccccaagtcct	accagacggaagatgaatcg
Bnip3	acccacagctttggtgagaa	cgcttgtgtttctcatgctg
Dusp1	ccatctgccttgcttacctc	tgatggggctttgaaggtag
FasL	ctgaaaccaaaaagccaagg	ctggctgttgcaagactgac
Gapdh	agaacatcatccctgcatcc	gtcctcagtgtagcccagga
IL6	agttgccttcttgggactga	acagtgcatcatcgctgttc
Nr4a2	cccagtggagggtaaactca	ccactctcttgggttccttg
Nurr77	tgttgctagagtccgccttt	cagtgatgaggaccagagca
Puma	cgtgtggaggaggaggagt	tagttgggctccatttctgg
Rgs2-F	gaggagaagcgggagaaaat	gaatgcagcaagcccatatt
spliced Tnf-α	actcgagtgacaagcccgta	gtgggtgaggagcacgtagt
Total Tnf-α	ccgatttgccatttcatacc	ccggactccgtgatgtctaa
Trail	ggatcactcggagaagcaac	agcctccttgaaccggtagt

Supplementary Movie 1. Astrocyte in the glial lamina of 10-month-old DBA/2J-*Gpnmb*$^{+}$ mice presents an irregular arrangement of processes, which is likely a shape of hook and loop.

Supplementary Movie 2. Astrocyte in the glial lamina of 10-month-old glaucomatous DBA/2J mice presents a loose arrangement of astrocytic processes caused by moderate glaucoma damage.

Supplementary Movie 3. Astrocyte in the glial lamina of 10-month-old glaucomatous DBA/2J mice presents a significant loss of astrocytic processes caused by severe glaucoma damage.
